# Altered Impedance of Ear Acupuncture Point MT2 in Breast Cancer Patients: A Preliminary Observation

**DOI:** 10.1155/2015/909246

**Published:** 2015-10-04

**Authors:** Yine Hu, Huayuan Yang, Pin Wang, Tangyi Liu, Wenchao Tang

**Affiliations:** ^1^Laboratory of Traditional Chinese Medicine Engineering, Shanghai University of Traditional Chinese Medicine, 1200 Cailun Road, Shanghai 201203, China; ^2^Anhui University of Traditional Chinese Medicine, 103 Meishan Road, Hefei 230038, China

## Abstract

Skin impedance at acupuncture points (APs) has been used as a diagnostic aid for more than 50 years. In this study, we have a diagnostic tool (JXT-2008) to measure the skin impedance of ear APs of 30 breast cancer patients and the corresponding skin impedance of ear APs of 30 healthy humans, and then we compared these changes in ear AP impedance in breast cancer patients and healthy individuals.

## 1. Introduction

Acupuncture is a discipline in traditional Chinese medicine, which includes the practice of inserting fine needles into the skin at specific anatomical locations to stimulate certain points on the body. This therapeutic strategy has been practiced for thousands of years in the treatment of various human diseases. Acupuncture has already been adopted by a number of countries as a type of complementary or alternative medicine in the treatment of certain human diseases [1]. The general principle of acupuncture, based on traditional Chinese medicine, is that stimulation of particular acupuncture points (APs) can correct the imbalances of the flow of Qi through specific channels known as meridians [2]. Qi in Chinese traditional medicine is used to describe the refined nutritious substance that constitutes the human body and maintains life activities, such as Gu-Qi and nutrient Qi. Qi is also used to describe functions of Zang-Fu organs, such as heart Qi and liver Qi [3]. However, to date, scientific research has been unable to identify any histological or physiological associations between Qi and acupuncture points [4]. Acupuncture is specifically useful in the treatment of autonomic dysfunction, neurological conditions (such as migraine or pain), cardiovascular diseases, pulmonary diseases (such as asthma), drug abuse, psychological disorders, and others [5]. Thus, novel stimulation techniques and the discovery of new acupuncture points could lead to better acceptance by patients and more effective treatment of human diseases. To this end, a Japanese researcher claimed that the presence of low skin impedance at acupuncture points is better than that of the non-APs [6]. Thereafter, an instrument was developed to detect electric reaction in skin as a diagnostic aid in Germany [7]. Earlier studies on the skin impedance were particularly useful in the diagnosis and treatment of different diseases. Studies have shown that APs have been shown to have lower skin impedance than the non-APs in the healthy individuals [8–10] and therefore changes in skin impedance at APs can be of diagnostic significance. However, another study showed that skin impedance at APs can either be lower or higher compared to the surrounding skin area in the healthy individuals; thus, electrical skin resistance measurement may not be used for acupuncture point localization or diagnostic purposes [11]. This observation could be due to the fact that these studies included healthy individuals only. Another study indeed reported that there was no difference between APs and non-APs in healthy individuals and changes may occur during illness [12]. Thus, we assessed and detected the skin impedance between the healthy individuals and the cancer patients in this study. However, according to traditional Chinese medicine, acupuncture meridians are believed to constitute channels for Qi flow connecting the surface of the body to internal organs. The theory regarding acupuncture meridians as “transmission lines” suggested that meridians may represent networks of preferential electrical current flow [13].

Study on the skin impedance in the ear's APs was conducted as an early diagnosis strategy for the detection of certain cancer in patients. In the presence of benign or malignant tumor in the body, the meridians may sense and react to the meeting point of the body surface and internal organs, and then a series of changes in biological and physical activities will appear on the surface of the ear [14]. A study investigating AP reaction in a total of 745 patients with diseases in the digestive system, respiratory system, and cardiovascular system diagnosed by western medicine revealed positive association between diagnoses made by western medicine and APs [15]. The purpose of this study was to use a nonlinear detecting method to detect impedance in different ear's APs, to observe their basic features, and finally to compare LIA, LDA, RIA, and RDA to determine skin resistance. In addition, we also assessed whether ear skin impedance at APs can be used as a diagnostic tool for breast cancer. The observation will provide evidence on whether there are differences in skin impedances at APs in the same ear between breast cancer patients and healthy subjects.

## 2. Materials and Methods

### 2.1. Experimental Device

**Figure 1:**
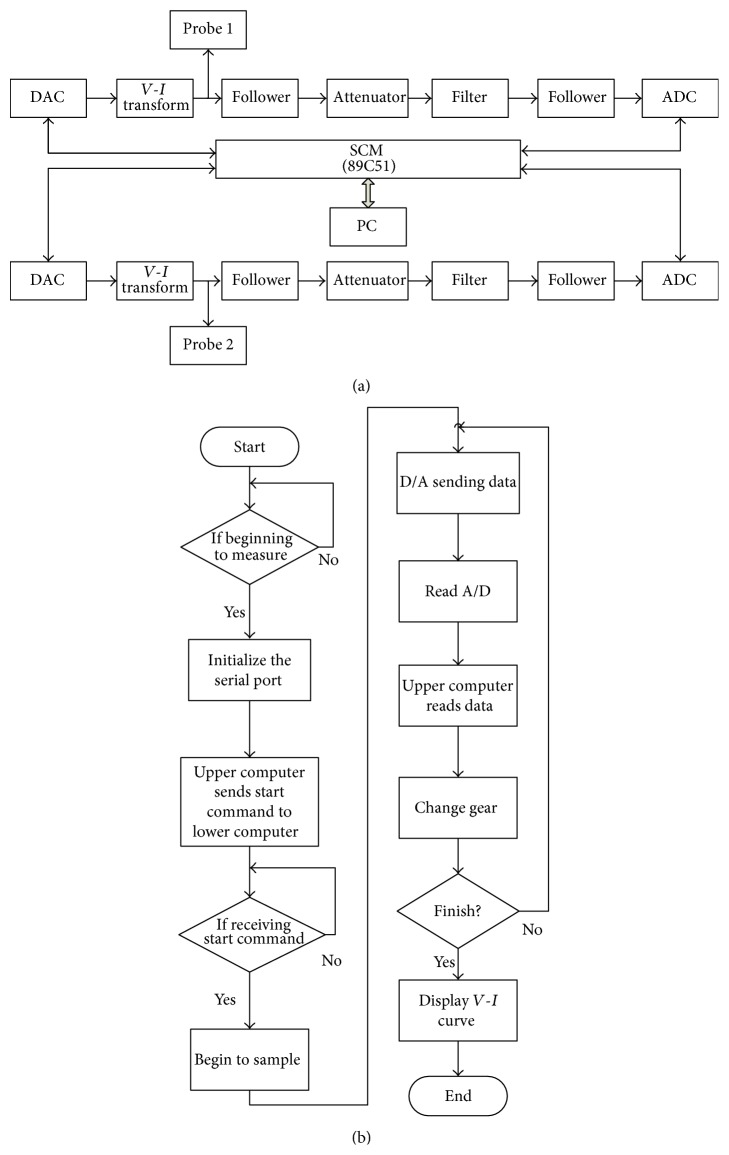
Illustration of our measurement device. (a) Hardware schematic diagram of the skin impedance detection system (JXT-2008). (b) Software schematic diagram of the skin impedance detection system (JXT-2008).

To detect changes in impedance at APs on the ear for the detection of breast cancer, we utilized the skin impedance detection system (model: JXT-2008) that was developed by Shanghai University of Traditional Chinese Medicine (Shanghai, China). A sensitive skin impedance detection-analysis system was used and internal design principles are shown in Figure 1.

The impedance at ear APs was measured after ensuring that the patients have established ground contact and using the device (i.e., JXT-2008) the patients were provided with continuous current with increasing intensity from 0 *μ*A to 20 *μ*A and then returning back from 20 *μ*A to 0 *μ*A. This enabled the measurement of current-voltage characteristics curve on the surface of the human body. The system consists of 89C51 single-chip microcomputer (SCM), a digital-analog component (DAC), an analog-digital component (ADC), a constant current source component (CCS), a voltage follower, an attenuator, and two probes. The SCM was connected to a personal computer through USB data line. The hardware and software design of this skin impedance detecting instrument is illustrated in Figures 1(a) and 1(b), respectively.

### 2.2. Study Subjects

A total of 60 subjects (30 breast cancer patients and 30 healthy control individuals) were recruited from Longhua Hospital and Shanghai University of TCM (Shanghai, China) between August 2007 and January 2008. The 30 breast cancer patients recruited were all female aged between 23 and 45 years (with mean age of 34.6 ± 12.14 years). This study was approved by the Ethics Committee of Shanghai Society and all participants signed an informed consent to participate in this study.

#### 2.2.1. Inclusion Criteria

Inclusion criteria included (1) patients who were diagnosed with breast cancer histologically and (2) patients who had undergone complete radiotherapy and chemotherapy.

#### 2.2.2. Exclusion Criteria

Exclusion criteria included patients who have any serious cardiovascular and cerebrovascular diseases, diseases of the blood system, serious damage to the liver and kidney functions, mental illness, or other cancers.

All participants completed quality of life questionnaires or psychiatric evaluation. Furthermore, 30 healthy controls were also recruited from Shanghai University of TCM (Shanghai, China) and these female volunteers were between 18 and 38 years (with mean age of 28.3 ± 8.54 years). They were all “healthy” as defined by normal body temperature and with no known autonomic nervous system dysfunction, coronary heart disease, systemic disease, cancer, or skin disease [16].

**Figure 2:**
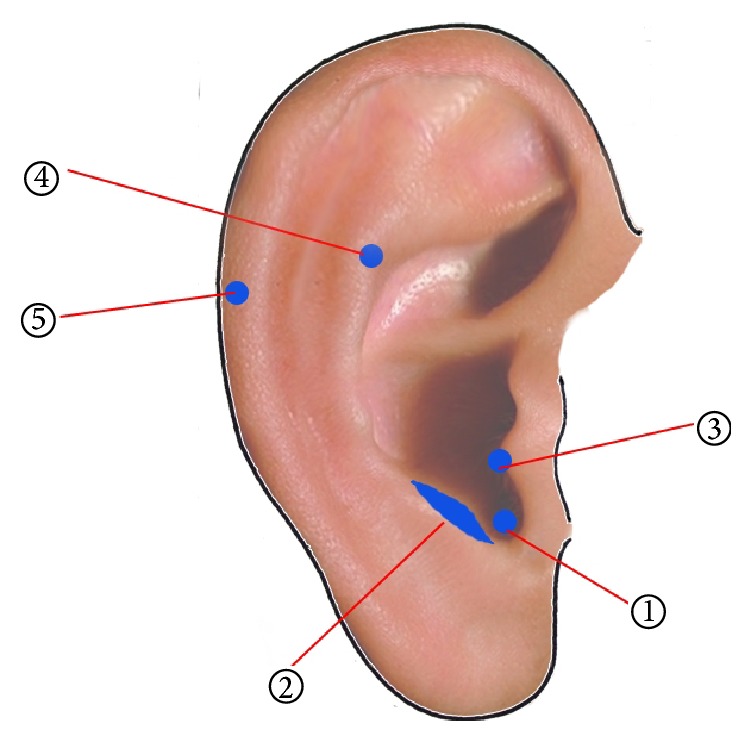
Ear APs location. ① CO18 (Neifenmi); ② AT4 (Pizhixia); ③ TG2p (Shenshangxian); ④ BP-B3 (Ruxian); and ⑤ MT2 (malignant tumor 2).

*Ear APs Selection and Localization*. A previous study demonstrated acupuncture treatment using the ear APs in the management of breast hyperplasia, and the ear APs were TF4 (Shenmen), BP-B3 (Ruxian), CO18 (Neifenmi), and AT4 (Pizhixia) [17]. In addition, the endocrine-related APs TG2p (Shenshangxian) [18] were also included in the current study. Moreover, clinical experience indicates that some specific area on the ear APs can provide a reference for the diagnosis of malignancies and these APs were named as MT1 (malignant tumor 1), MT2, and MT3, while MT2 is more closely associated with the diagnosis of malignancies [19]. Together, this study included the following ear APs (i.e., the bilateral CO18 (Neifenmi), AT4 (Pizhixia), TG2p (Shenshangxian), BP-B3 (Ruxian), and MT2 (malignant tumor 2)), which are more related to breast diseases. The localization of these ear APs was detected by GB/T13734-2008 nomenclature and location of ear points (Figure 2).

*Detection Methods*. The experiment was performed under quiet, controlled conditions with room temperature ranging between 20 and 26°C with a relative humidity of 50% to 75%. All subjects were asked to arrive at the laboratory more than 15 minutes prior to the experiment and sat quietly to relax their muscles. The skin impedance was measured after the ear skin was cleaned with 75% alcohol for 30 seconds. The detecting probe was placed on the points with 75 g/mm^2^ pressure with a detection angle of 90 degrees for a total scanning time of 20 seconds. The scanning electrical current was increased steadily and linearly from 0 *μ*A to 20 *μ*A in the first 10 seconds and was decreased steadily from 20 *μ*A to 0 *μ*A in the second 10 seconds. Measurements at every APs were recorded once every 3 seconds and the measurements cycle was repeated three times. Data were summarized as mean ± SD.

*Observation Index*. The quantitative index of the skin impedance of APs included Left Increase Area (LIA), Right Increase Area (RIA), Left Decrease Area (LDA), and Right Decrease Area (RDA). Increase Area (IA) is referred to as the area comprised of the current-voltage curve (currents intensity was increased from 0 *μ*A to 20 *μ*A) and abscissa, which was calculated by integral calculus. LIA refers to the left AP of two bilateral symmetrical APs, and RIA refers to the right one. When currents intensity was reversed from 20 *μ*A to 0 *μ*A, the corresponding parameters (LDA and RDA) were obtained. The area was calculated according to the following formula:

(1) Increase  Area=∫020udI,Decrease  Area=∫200udI.

*Statistical Analysis*. Impedance in each ear's AP was measured 3 times and the data were summarized as mean ± SD. Two independent samples' *t*-test was applied to compare the differences between healthy controls and breast cancer patients. The SPSS 16.0 statistical software (SPSS, Chicago, IL) was used for statistical analysis and *P* value less than 0.05 was considered to be statistically significant.

## 3. Results

A total of 10 APs (i.e., bilateral CO18 (Neifenmi), AT4 (Pizhixia), TG2p (Shenshangxian), BP-B3 (Ruxian), and MT2 (malignant tumor 2)) were measured in 30 healthy individuals (the control group) and 30 breast cancer patients. The data are shown in Tables 1–5. Our results indicate that the skin impedance measured in LIA, LDA, RIA, and RDA of bilateral ear APs (AT4 and TG2p) was found to be higher in patients than those of the healthy individuals (control group); however, the differences were not statistically significant (*P* > 0.05) (Tables 2 and 3). The AP spots of CO18 and BP-B3 recorded similar results; that is, at ear APs, CO18 and BP-B3, the impedances in LIA and LDA were higher in patients than those of the healthy individuals (control group), whereas the RIA and RDA were lower in patients than those of the healthy individuals (control group), but the differences were not statistically significant (*P* > 0.05) (Tables 1 and 4). Interestingly, volt-ampere area at AP MT2 (malignant tumor 2) was significantly (*P* < 0.05) higher in patients than those of the healthy individuals. Skin impedance of the ear AP MT2 in breast cancer patients was significantly increased (LIA, *P* value = 0.0047; LDA, *P* value = 0.0028; RIA, *P* value = 0.0030; RDA, *P* value = 0.0015) as listed in Table 5. Statistical analyses indicate that all detected ear APs had nonlinear characteristics in both healthy control individuals and breast cancer patients. The skin impedance at other ear APs (i.e., CO18, AT4, TG2p, and BP-B3) was also altered in breast cancer patients, but the changes were not statistically significant compared to the healthy controls (*P* > 0.05).

## 4. Discussion

In the current study, we measured the ear APs impedance in breast cancer patients and healthy controls and found that the ear APs impedance in breast cancer patients can be either lower or higher compared to that of the healthy controls. The alterations in ear APs impedance could aid in diagnosis of these patients. If the APs skin impedance was regarded as a “point” that changes in time and space, then the volt-ampere area (i.e., LIA, LDA, RIA, and RDA) reflects comprehensive dynamic resistance between APs. Specifically, we identified that in LIA, LDA, RIA, and RDA and there was a statistically significant difference in bilateral ear AP MT2 between breast cancer patients and healthy individuals. Scientific evidence suggested that ear skin impedance at the APs is electrically distinct from non-AP sites and that changes in ear skin AP impedance could be of substantial diagnostic, therapeutic, and research significance. Indeed, in China and certain western countries, different researchers have tried to demonstrate a difference between the skin impedance at AP and the surrounding impedance and showed that APs had lower skin resistance than nearby non-AP sites [20, 21], which is useful in localization of APs. Moreover, another study showed that skin impedance at AP sites could be either lower or, in contrast to common belief, higher compared to the surrounding area and that this phenomenon had a high short-term and a low long-term reproducibility. It is important to note that these data were obtained from healthy individuals, and there were no data on skin impedance at AP sites in individuals suffering from a disease.

Evidence based on previous studies indicated that in certain diseases higher or lower resistance of skin impedance at APs was associated with clinically relevant APs; for example, in stomach diseases, the APs with differential impedance were observed in ST36 (Zusanli), RN12 (Zhongwan), and BL21 (Weishu), but not at other clinically unrelated APs or non-AP sites in the same patients [22, 23]. Observations from the current study were consistent with a previous study that higher value at the volt-ampere area indicated greater impedance and the nonlinear characteristics of the volt-ampere curve [24]. Our earlier studies with this skin impedance measuring device reported skin impedances in stroke patients were higher than that of the healthy controls [25, 26].

As a result, skin impedance value may be used in the early aid diagnosis of breast cancer. It was suggested that its changes could be due to loss of cellular cohesion and increase in the extracellular space in skin tumors [27]. According to Sugar's report, the extracellular spaces during inflammation also increased, but the increase was significantly less than in cancerous tissues. Thus, it is possible that skin impedance value may be appropriate for the early detection of different pathologies. In our current study, we selected five clinically relevant APs (bilateral CO18, AT4, TG2p, BP-B3, and MT2). We found that two clinically related APs (bilateral MT2) of breast cancer patients had higher skin impedance than that of the healthy controls, consistent with a previous study that characterized a slight increase of the normalized resistance in breast cancer patients versus healthy women [28]. However, our data was on impedance at APs on ear skin, based on the traditional acupuncture meridians theory of traditional Chinese medicine. An earlier study using impedance measurement system reported by Keshtkar et al. also showed difference in impedance between normal and malignant bladder tissue [29] and the resistivity of malignant tissues was higher than that of the nonmalignant tissues. However, our current data on ear APs (i.e., CO18, AT4, TG2p, and BP-B3) were not similar to those reported by Keshtkar et al. The skin impedance values on the left or the right APs often vary, the skin impedance value of the same side of viscera that suffered from an illness was much lower than that of the side that was healthy, and this was very meaningful to locate the lesion in human body, which is in accordance with the meridian balance theory. Use of the APs as a diagnostic tool is carried out based on the understanding that APs have an association with the internal organs of the human body. The modern electronic technology combined with traditional Chinese medicine diagnostic method was used widely in different types of scientific research [30, 31]. The skin impedance measurement of the APs gave an enormous impetus to development of diagnostic tools in traditional Chinese medicine, which can help to show certain information about the pathological changes more deeply and comprehensively.

To the best of our knowledge, there are no studies in scientific literature describing skin impedance at the same ear APs between breast cancer patients and healthy controls. We found that ear APs of breast cancer patients and healthy individuals might possess different specific electrical properties. Since the majority of the measured ear APs did not show higher skin impedance, it cannot be concluded from our data that the skin impedance measurements of ear APs (i.e., CO18, AT4, TG2p, and BP-B3) can be used for diagnostic purpose. However, the ear AP MT2 site could be used as diagnostic pointer in diagnosis of breast cancer in patients. Nevertheless, a possible limitation of this study is that it is based on a single population and the observations made may not be applicable for another population. The impedance did differ significantly between older and younger people in both the control and patient groups. However, it is important to note that the age of the study population was 45 years or younger.

## 5. Conclusions

The current study demonstrates that the skin impedance of breast cancer patients was lower than that of healthy controls only at one AP (MT2) in the ear and there were no differences in the impedance at other ear APs between the groups. Further studies involving a larger cohort are necessary to verify the current observation and investigate its accuracy in the detection of early stage breast cancer and staging of the disease.

## Figures and Tables

**Table 1:** Comparison of LIA, LDA, RIA, and RDA between patients and controls in CO18.

Index	Groups	Number	Area ($\overset{-}{X}\pm S$)	*P* value	*t* value
LIA	Breast cancer	30	57.20 ± 41.09	0.39	0.58
	Control	30	52.04 ± 26.03		

LDA	Breast cancer	30	52.20 ± 39.16	0.53	0.84
	Control	30	45.30 ± 22.39		

RIA	Breast cancer	30	39.90 ± 18.27	0.33	0.59
	Control	30	43.29 ± 25.60		

RDA	Breast cancer	30	36.16 ± 15.66	0.48	0.62
	Control	30	39.23 ± 22.24		

**Table 2:** Comparison of LIA, LDA, RIA, and RDA between patients and controls in AT4.

Index	Groups	Number	Area ($\overset{-}{X}\pm S$)	*P* value	*t* value
LIA	Breast cancer	30	85.74 ± 46.44	0.64	0.46
	Control	30	80.78 ± 36.96		

LDA	Breast cancer	30	76.94 ± 43.31	0.82	0.62
	Control	30	70.84 ± 32.89		

RIA	Breast cancer	30	70.15 ± 35.80	0.65	0.26
	Control	30	67.87 ± 33.04		

RDA	Breast cancer	30	62.31 ± 31.24	0.77	0.23
	Control	30	60.54 ± 29.45		

**Table 3:** Comparison of LIA, LDA, RIA, and RDA between patients and controls in TG2p.

Index	Groups	Number	Area ($\overset{-}{X}\pm S$)	*P* value	*t* value
LIA	Breast cancer	30	68.70 ± 43.80	0.49	1.06
	Control	30	58.15 ± 32.40		

LDA	Breast cancer	30	59.69 ± 39.20	0.42	1.10
	Control	30	49.96 ± 28.39		

RIA	Breast cancer	30	57.86 ± 39.19	0.43	0.43
	Control	30	54.03 ± 28.13		

RDA	Breast cancer	30	50.08 ± 33.65	0.38	0.35
	Control	30	47.37 ± 24.58		

**Table 4:** Comparison of the LIA, LDA, RIA, and RDA between patients and controls in BP-B3.

Index	Groups	Number	Area ($\overset{-}{X}\pm S$)	*P* value	*t* value
LIA	Breast cancer	30	123.95 ± 45.74	0.11	1.58
	Control	30	105.34 ± 45.55		

LDA	Breast cancer	30	109.15 ± 43.22	0.20	1.29
	Control	30	94.85 ± 42.92		

RIA	Breast cancer	30	103.05 ± 46.15	0.77	0.28
	Control	30	106.44 ± 46.99		

RDA	Breast cancer	30	92.46 ± 42.62	0.91	0.11
	Control	30	93.66 ± 42.33		

**Table 5:** Comparison of LIA, LDA, RIA, and RDA between patients and controls in MT2.

Index	Groups	Number	Area ($\overset{-}{X}\pm S$)	*P* values	*t* values
LIA	BC patients	30	79.64 ± 47.79^*∗∗*^	0.0047	3.42
	Control	30	47.36 ± 19.77		

LDA	BC patients	30	70.47 ± 43.45^*∗∗*^	0.0028	3.32
	Control	30	41.84 ± 18.60		

RIA	BC patients	30	81.40 ± 46.91^*∗∗*^	0.0030	3.31
	Control	30	49.50 ± 24.18		

RDA	BC patients	30	71.12 ± 41.20^*∗∗*^	0.0015	3.52
	Control	30	41.81 ± 19.49		

^*∗∗*^ *P* < 0.01.

## Abbreviations

Bilateral CO18: Neifenmi
AT4: Pizhixia
TG2p: Shenshangxian
BP-B3: Ruxian
MT2: Malignant tumor 2
APs: Acupuncture points
JXT-2008: JinXueTanCe-2008.
